# *Anacyclus pyrethrum* var. *pyrethrum* (L.) and *Anacyclus pyrethrum* var. *depressus* (Ball) Maire: Correlation between Total Phenolic and Flavonoid Contents with Antioxidant and Antimicrobial Activities of Chemically Characterized Extracts

**DOI:** 10.3390/plants10010149

**Published:** 2021-01-13

**Authors:** Fatima Zahra Jawhari, Abdelfattah E. L. Moussaoui, Mohammed Bourhia, Hamada Imtara, Hamza Saghrouchni, Kenza Ammor, Hayat Ouassou, Youssef Elamine, Riaz Ullah, Essam Ezzeldin, Gamal A. E. Mostafa, Amina Bari

**Affiliations:** 1 Laboratory of Biotechnology, Environment, Agri-Food, and Health (LBEAS), Faculty of Sciences, University Sidi Mohamed Ben Abdellah (USMBA), Fez 30050, Morocco; abdelfattah.elmoussaoui@usmba.ac.ma (A.E.L.M.); amina.bari@usmba.ac.ma (A.B.); 2 Laboratory of Chemistry-Biochemistry, Environment, Nutrition, and Health, Faculty of Medicine and Pharmacy, University of Casablanca, B.P. 5696, Casablanca 20250, Morocco; 3 Faculty of Arts and Sciences, Arab American University Palestine, P.O. Box 240, Jenin 44862, Palestine; hamada.tarayrah@gmail.com; 4 Department of Biotechnology, Institute of Natural and Applied Sciences, Çukurova University, 1380 Adana, Turkey; hsaghrouchni@student.cu.edu.tr; 5 Laboratory of Materials Engineering and Environment, Faculty of Sciences, University Sidi Mohamed Ben Abdellah (USMBA), Fez 30050, Morocco; kenza.ammor1@gmail.com; 6 Laboratory of Physiology, Genetics, and Ethnopharmacology, Department of Biology, Faculty of Sciences, Mohammed First University, B.P. 524, Oujda 60000, Morocco; Hayat.Ouassou@ump.ac.ma; 7 Laboratory Physiology-Pharmacology & Environmental Health, University Sidi Mohamed Ben Abdallah (USMBA), Fez 30050, Morocco; elamineysef@gmail.com; 8 Department of Pharmacognosy, College of Pharmacy, King Saud University, Riyadh 11451, Saudi Arabia; rullah@ksu.edu.sa; 9 Department of Pharmaceutical Chemistry, College of Pharmacy King Saud University, Riyadh 11451, Saudi Arabia; esali@ksu.edu.sa (E.E.); gmostafa@ksu.edu.sa (G.A.E.M.); 10 Micro-Analytical Laboratory, Applied Organic Chemistry Department, National Research Center, Dokki, Cairo 12622, Egypt

**Keywords:** mineral analysis, phytochemical analysis, antibacterial activity, antioxidant activity, *Anacyclus pyrethrum* var. *pyrethrum* (L.), *Anacyclus pyrethrum* var. *depressus* (Ball) Maire

## Abstract

In this work, two varieties of *Anacyclus pyrethrum* (L.) including *Anacyclus pyrethrum* var. *pyrethrum* (L.) and *Anacyclus pyrethrum* var. *depressus* (Ball) Maire were evaluated for their mineral and chemical compositions, total phenolic and flavonoid contents, and antimicrobial and antioxidant activities using hydroalcoholic extracts from their different parts (leaves, capitula, roots, and seeds). The phytochemical and mineral compositions were carried out using standard methods. The antioxidant activity was determined using the DPPH (2,2-diphenyl-1-picrylhydrazyl), ABTS (2,2-azino-bis 3-ethylbenzothiazolin-6-sulfonic acid), and FRAP (ferric reducing antioxidant power) tests. The antimicrobial activity was assayed using the agar diffusion, minimum inhibitory concentration, and minimum bactericidal concentration methods. The results of the chemical analysis showed that both varieties contained interesting mineral and chemical compositions with potentially active compounds; among them, *N*-isobutyl-2,4-heptadiene-6-monoynamide and cinnamic acid were detected in the *Anacyclus pyrethrum* var. *pyrethrum* (L.) only while thiadiazolo [5,4-d] pyrimidin-7-amine and *N*-isobutyl-2,4-undecadiene-8,10-diynamide compounds were limited to the *Anacyclus pyrethrum* var. *depressus* (Ball) Maire. In vitro antioxidant and antimicrobial activities of the two varieties demonstrated that the different parts had prominent antioxidant and antimicrobial properties. The principal component analysis (PCA) showed great similarity in the activity of the leaves, capitula, and seeds of both plants and a high difference in roots. *Anacyclus pyrethrum* var. *pyrethrum* roots were characterized by a high content in phenols and flavonoids and better antibacterial activities compared to *Anacyclus pyrethrum* var. *depressus* (Ball) Maire roots, which were characterized by better antioxidant activities. From this study, it can be concluded that the two varieties of *Anacyclus pyrethrum* (L.) showed promising mineral and chemical compositions with antioxidant and antimicrobial properties.

## 1. Introduction

Knowledge of the healing properties of medicinal plants has been transmitted over the centuries within and among human communities. Bioactive compounds produced in plant species are usually responsible for their pharmacological properties such as antidiabetic, anti-inflammatory, anticancer, and antimicrobial activities [1,2]. Bacteria, fungi, and viruses are responsible for causing many infectious diseases [3]. Antimicrobial resistance has been observed with time, even though many modern antimicrobial drugs have been developed to manage contagious diseases. Oxidative stress occurs from the imbalance between the production of reactive species, and antioxidant defense activity is involved in several chronic diseases including cancer, diabetes, cardiovascular diseases, chronic kidney disease, and neurodegenerative diseases [4]. Even though several studies have explored the antimicrobial and antioxidant effects of natural compounds, a huge number of plants have not been investigated for their potential activities [5,6].

*Anacyclus pyrethrum* (L.) (*A. pyrethrum* (L.)) is a species belonging to the Asteraceae family. This species includes two varieties *Anacyclus pyrethrum* var. *depressus* (Ball) Maire (*A. P* var. *depressus* (Ball) Maire) and *Anacyclus pyrethrum* var. *pyrethrum* (*A. P* var. *pyrethrum* (L.)) [7,8], which grow in Morocco, Algeria, and Spain [9,10]. The two varieties differ morphologically by the size of the flower heads, the length of the roots, and the color of the petal back [7,8]. As reported in earlier data, the roots of *A. pyrethrum* (L.). possess interesting pharmacological properties including anticancer [11], aphrodisiac [12,13,14], anti-convulsive [15], androgenic and fertilizing [12,16], antiparasitic and antibiotic [17], bio-insecticide [18], anti-amnesic [19], antidiabetic [20,21], antifungal, and immunostimulant effects [16,22]. In addition, this plant is prescribed for treating partial paralysis of the tongue and lips [23], gout, and sciatica [24].

To the best of our knowledge, no previous literature has investigated the pharmacological activities of the two varieties *A. P* var. *depressus (Ball) Maire and A. P* var. *pyrethrum.* (L.). It is thus fitting that we study for the first time the mineral and the chemical compositions and the antimicrobial and antioxidant activities of different parts from the two varieties. The antimicrobial activity was studied using the agar diffusion, Minimum Inhibitory Concentration (MIC), and Minimum Bactericidal Concentration (MBC) methods. The antioxidant activity was studied using three in vitro assays: inhibition of the 2,2-diphenyl-1-picrylhydrazyl (DPPH) radical, the ferric reducing antioxidant power (FRAP), and ABTS+ (2,2-azino-bis(3-ethylbenzthiazoline-6-sulfonic acid) free radical scavenging activity. The phytochemical, mineral, total phenolic, and flavonoid contents of hydroalcoholic extracts were determined as described within this study. Data in its entirety were used to study the correlations between the evaluated parameters and to run the principal component analysis (PCA) for discrimination of the samples.

## 2. Results and Discussion

### 2.1. Extraction Yield

The yield (appearance and color) of each extract is represented in Table 1. The results showed that the hydro-ethanol extracts of different parts of *A. pyrethrum* (L.) varieties have two aspects: pasty and powdery with two different colors (green and brown). The maximum yield was obtained from *A. P* var. *pyrethrum* root extracts (16%), followed by the leaves and capitula extracts (14%), while the seeds presented the lowest yield (10%). In contrast, the extraction from *A. P* var. *depressus* (Ball) Maire presented the lowest yield (capitula: 6%; roots: 13%).

### 2.2. Phytochemical Characterization of Plant Extracts

**Figure 1:**
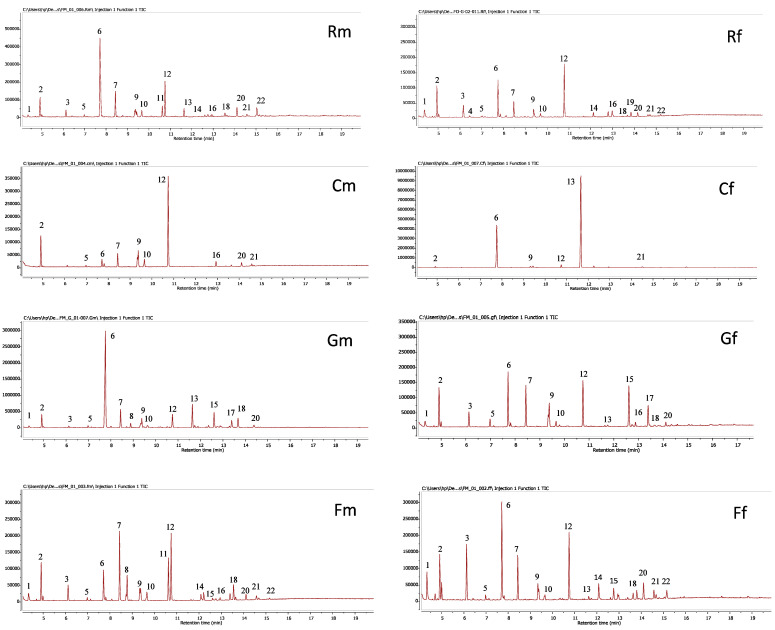
Chromatographic profile of different parts (roots, seeds, leaves, and capitula) of the two varieties *A. P* var. *pyrethrum* (L.) and *A. P* var. *depressus* (Ball): Rm: roots of. *P* var. *pyrethrum* (L.); Gm: seeds of *A. P* var. *pyrethrum* (L.); Fm: leaves *A. P* var. *pyrethrum* (L.); Cm: capitula of *A. P* var. *pyrethrum* (L.); Rf: roots of *A. P* var. *depressus* (Ball) Maire; Gf: seeds of *A. P* var. *depressus* (Ball) Maire; Ff: leaves of *A. P* var. *depressus* (Ball) Maire; Cf: capitula of *A. P* var. *depressus* (Ball) Maire.

The extracts of different parts (roots, seeds, leaves, and capitula) of *A. P* var. *pyrethrum* (L.) and *A. P* var. *depressus* (Ball) Maire were analyzed by GC-MS after silylation. As shown in Table 2 and Figure 1, GC-MS analysis confirmed the presence of twenty-two compounds in the extracts studied. The results of the chemical analysis show that the two varieties share the same compounds except for the *N*-isobutyl-2,4-heptadiene-6-monoynamide and cinnamic acid compounds, which were detected in *A. P* var. *pyrethrum* only. The detection of thiadiazolo [5,4-d] pyrimidin-7-amine and *N*-isobutyl-2,4-undecadiene-8,10-diynamide compounds were limited to *A. P* var. *depressus* (Ball) only. Indeed, the chemical analysis of *A. P* var. *pyrethrum* extracts revealed the presence of *N*-isobutyl-dodeca-2,4,8,10-tetraenamide; *N*-isobutyl-2,4-octadiene-6-monoynamide; levulinic acid; malonic acid; palmitic acid; morphinan-6-one; 4,5α-epoxy-3-hydroxy-17-methyl; 2,4-undecadiene-8,10-diyne-*N*-tyramide; dodeca-2E,4E; nE-trienoic acid; and 4-hydroxyphenylethylamide compounds in all parts studied (roots, seeds, leaves, and capitula), whereas the variety *A. P* var. *depressus* was distinguished by the presence of *N*-isobutyl-dodeca-2,4,8,10-tetraenamide; levulinic acid; palmitic acid; and 2,4-undecadiene-8,10-diyne-*N*-tyramide compounds. Chromatographic analysis of the studied extracts affirmed the presence of several components in the capitula, leaves, and seeds of *A. P* var. *pyrethrum* (L.) and *A. P* var. *depressus* (Ball) that may be involved in antioxidant and antibacterial activities.

In the present work, twenty-two compounds were detected by GC-MS after silylation, and among them, propanedioic acid; levulinic acid; sarcosine, *N*-(trifluoroacetyl)-butyl ester; morphinan-6-one; 4,5α-epoxy-3-hydroxy-17-methyl; palmitic acid; isovaleric acid; and 2,4-undecadiene-8,10-diyne-*N*-tyramide compounds were newly detected, to the best of our knowledge. Some of the compounds identified in the studied extracts of *A. P* var. *pyrethrum* (L.) and *A. P* var. *depressus* (Ball), such as pellitorine, are antibacterial, insecticidal, anticoagulant, anticancer, and anti-inflammatory agents [25,26,27]. Alkylamides (*N*-isobutyl-2,4-octadiene-6-monoynamide; (2,4)-*N*-isobutyl-2,4-undecadiene-8,10-diynamide; *N*-isobutyl-2,4-heptadiene-6-monoynamide; *N*-isobutyl-dodeca-2,4,8,10-tetraenamide; and *N*-isobutyl-2,6,8-datrienamide) were previously confirmed to have activities, such as antioxidant, antimicrobial, anticancer, antithrombotic, antiviral, anti-inflammatory, immunomodulatory, analgesic, antiprotozoal, and antidiabetic activities [13,28,29,30]. Moreover, propanedioic acid was found to be a monoamine oxidase inhibitor agent with antimycobacterial, antimicrobial, antiviral, anti-HIV, anticancer, antiparasitic, anticonvulsant, antidiabetic, antihypertensive, and anti-hyperlipidemic activities [31]. Cinnamic acid is well-known for its antioxidant, antimicrobial, antitumor, and antimycobacterial properties [32,33,34]. Moreover, cinnamic acid possesses many biological activities, as reported by Hafizur et al. (2015), stating that this compound exhibited antidiabetic activity by decreasing blood glucose levels and by improving glucose tolerance in diabetic rats in a time- and dose-dependent manner. The cinnamic acid enhanced glucose-stimulated insulin secretion in isolated islets [35] over its antibacterial activity against *A. sobria, A. salmonicida, L. anguillarum, V. crassostreae,* and *Y. ruckeri* bacteria [36]. Levulinic acid was found to have antioxidant, anticonvulsant, and anti-inflammatory activities [37,38,39,40]. The mechanism of action of levulinic acid in bacteria inhibition was also investigated in previous literature [41], which showed that levulinic acid reacts in synergy with sodium dodecyl sulfate (SDS) to reduce the cytoplasmic pH of microbes by ionization of undissociated acid molecules, affects the electrostatic charge of SDS molecules and cell surfaces, disrupts substrate transport, reduces proton motive force, chelates metal ions, and thereby induces the release of lipopolysaccharides from the outer membrane of gram-negative bacteria. Isovaleric acid has a therapeutic effect as an anti-dyslipidemic anticonvulsant [42,43,44]. Morphinan-6-one and 4,5α-epoxy-3-hydroxy-17-methyl have analgesic activities [45,46]. Each component may represent different mechanisms of action, whether it reacts individually or synergizes with another, ultimately resulting in many effective therapeutic properties of extracts [47]. The richness of the studied extract in different chemical compounds with numerous activities as reported in the earlier literature could justify the results obtained in terms of antioxidant and antibacterial activities as described in the present work.

### 2.3. Mineral Analysis

The findings of mineral analysis showed that the studied plants were higher in oligo-elements, especially calcium, potassium, magnesium, iron, and phosphorus. Therefore, these results can justify the use of these plants as food supplements [11,15,18,24,48]. The results of the mineral analysis of different parts (leaves, capitula, roots, and seeds) of the two varieties are presented in Table 3. The findings obtained showed that calcium was predominant in leaves over the other elements detected (Bi, Cu, Fe, K, Mg, Mn, Na, P, Se, and Zn) while seeds had high levels of copper and iron. Particularly, leaves of *A. P* var. *pyrethrum* were higher in phosphorus content. Therefore, the two studied varieties were found to be different in terms of mineral concentrations.

### 2.4. Antioxidant Content

Polyphenols have a very important role in determining the biological activities of natural products. Their contents are largely impacted by environmental conditions like harvest period, extraction solvent, and storage conditions [49]. Our findings showed that the hydro-alcoholic extracts of the two varieties (Fm and Ff) were higher in total phenolic and flavonoid compounds (Table 4). The total phenolic contents of extracts from *A. P* var. *pyrethrum* and *A. P* var. *depressus* (Ball) Maire leaves were 51.78 ± 0.49 and 38.75 ± 2.91 mg GAE/g, respectively. The total flavonoid contents in leaves were 13.53 ± 0.05 and 9.57 ± 0.02 mg QE/g, respectively. On the other hand, the hydro-alcoholic root extract had the lowest amount of total phenolic (25.96 ± 1.9 and 5.44 ± 0.21mg GAE/g, respectively) and flavonoid contents (0.88 ± 0.02 and 2.40 ± 0.02 mg, QE/g respectively). The hydro-alcoholic extracts of different parts of *A. P* var. *pyrethrum* were richer in total phenolic and flavonoid than those of *A. P* var. *depressus* (Ball) Maire. These findings were consistent with the results reported by Cherrat et al. [49], which showed that extracts from genera Anacyclus contained polyphenol and flavonoid contents. The results of polyphenol and flavonoid contents found in the present study were lower than those reported by Sujith et al. and Daoudi et al. [15,50]. The difference in polyphenol content may depend on several factors such as solvent, extraction conditions, harvest period, and environmental conditions [51].

### 2.5. Antioxidant Activity

Recent studies have shown that there is no single method of assessment of antioxidant activity as each antioxidant activity is quantitatively and accurately distinguished by its mechanism of action. The results obtained showed significant antioxidant activities of different parts of *A. P* var. *pyrethrum* and *A. P* var. *depressus* (Ball) Maire with an IC_50_ ranged from 0.03 to 1.19 mg/mL compared to butylated hydroxytoluene (BHT), ascorbic acid, and Trolox (IC_50_: 0.009, 0.019, and 0.003 mg/mL, respectively) (Table 5). Ascorbic acid, Trolox, and BHT were used as positive controls. Based on the antioxidant activity evaluated by the DPPH test, we found that the Ff sample was the most active with IC_50_ of 0.033 ± 0.001 mg/mL and that the Rm sample with IC_50_ 0.18 ± 0.005 mg/mL was the least active when compared to the other samples. These results were concordant with those of the ABTS test. The reducing power of the samples studied depends on the dose; the results showed that the Gf sample was the most reducing, whereas Rm had the lowest activity (1.19 ± 0.005 mg/mL). The Rm sample had the highest total antioxidant activity with a content of 708.74 ± 11.63 mg AAE/g, while the Cf sample had the lowest activity value (160.33 ± 10.23 AAE/g). These findings were in accordance with those reported in a work by Cherrat et al. investigating chemical content in the genera Anacyclus [49].

### 2.6. Antibacterial Activity

This work aimed also to evaluate the antibacterial activity of different extracts of *A. P* var. *depressus* (Ball) Maire and *A. P* var. *pyrethrum* (L.). All extracts studied showed significant antimicrobial activities against the tested strains. Therefore, we could confirm that the tested extracts possess antimicrobial bioactive molecules.

The antimicrobial activity of extracts from different parts of *A. P* var. *pyrethrum* (L.) and *A. P* var. *depressus* (Ball) Maire was recorded against five bacteria (Table 6). The inhibition zone diameters of extracts studied ranged from 5.5 to 15.65 mm, and the highest inhibition zone values against pathogens of medical importance such as *Pseudomonas aeruginosa, Staphylococcus aureus*, and *Klebsiella pneumonia* were 15.65, 15, and 15.3 mm, respectively.

Besides, analysis of the antibacterial power also revealed a variation not only according to the nature of the extract used but also from one strain to another. The Cm sample had interesting antimicrobial activities against all strains studied. The inhibition zone diameter recorded for this sample ranged from 7.5 to 15.65 mm. Likewise, the Cf sample inhibited growth of all strains except *E. coli57* that showed resistance to this extract. The Gm sample generated an inhibition zone diameter ranging from 5.65 to 10 mm on *E. coli97* and a value ranging from 5.65 to 6.35 mm in diameter for *Pseudomonas aeruginosa*. The Gf sample had an inhibition zone of 8.5 mm against *E. coli97*, and inhibition zones of 7, 7.5, 7.5, and 7.8 mm were observed for *staphylococus, Klebsiella*, and *pseudomonas*, respectively. Meanwhile, *E. coli57* resisted this extract. The Ff sample was active on *E. coli97, pseudomona,* and *Klebsiella* with inhibition zones ranging from 5.5 to 11 mm; Fm was more active on *Klebsiella pneumonia*, with an inhibition diameter of 15.3 mm than other strains. The Rm was active on *E. coli97*, *pseudomonas,* and *Klebsiella* with inhibition zone diameters of 6.6, 5.5, and 6.1 mm, respectively. Rf had a bacterial growth inhibitory effect on *staphylococcus, pseudomonas,* and *Klebsiella* with values ranging from 5.75 to 11 mm. Meanwhile, *A. P* var. *depressus* (Ball) Maire extracts (Cf, Gf, Ff, and Rf) were inactive against *E. coli57.*

The results of MICs showed that all extracts revealed a positive result against the tested pathogens. Regarding the MBC results obtained after 24 h of bacterial growth, all extracts tested were found to have a relatively bactericidal effect with concentrations ranging from 3.125 to 200 mg/mL (Table 6).

The best activity was obtained by the extracts Rf and Rm; these effects were observed on *Staphylococcus aureus* with MIC values of 0.39 and 0.78, respectively. These results were in accordance with those reported in earlier works on the antimicrobial potency of plant extracts studied [17,52,53,54,55,56,57]. The present results consequently confirm the traditional use of *A. pyrethrum* (L.) against various infections. The antimicrobial power of essential oils from the roots of *A. pyrethrum* (L.) against *E. coli, S. aureus, P. aeruginosa*, and *K. pneumonia* was confirmed elsewhere [17].

Other studies showed that the essential oil of the Algerian genera Anacyclus had an interesting antimicrobial effect against strains of *C. albicans,* which are considered very dangerous and very difficult to eliminate [22,55]. In our study, an activity against *S. aureus* was found in the 7-mm inhibition zone for 200 mg of the Rf extract, while the Rm extract was not active on this strain. It is very likely that an increase in the quantity of the extract could cause higher activity against this bacteria, as reported in an earlier work [54].

The MIC results reported in this study were superior to those reported in earlier works [17,52,54,55]. The difference between our study and previous studies might be for several reasons: the strains used may be different, and the location and collection season of two varieties of *A*. *pyrethrum* (L.) may cause a change in the active components.

### 2.7. Multivariable Analysis

**Figure 2:**
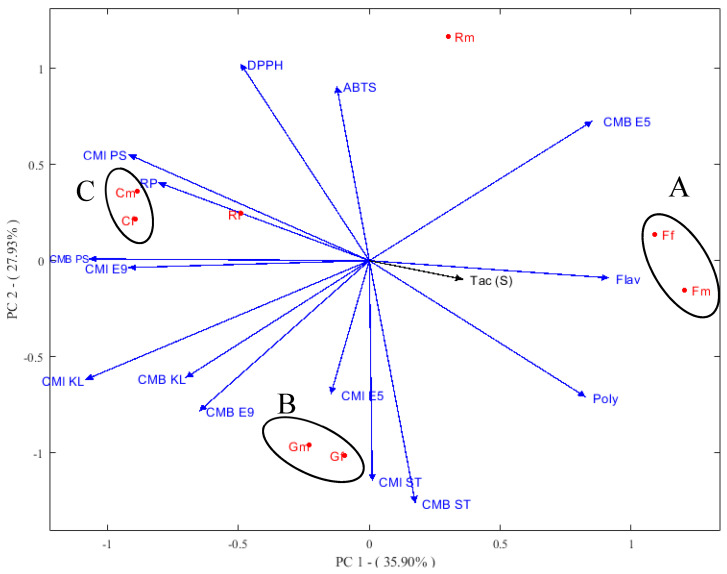
Analysis of the main components of antioxidant and antibacterial activities (poly: total polyphenol; Flav: flavonoid; TAC: total antioxidant capacity; DI: diameter inhibition; E5: *E. coliB6N*; E9: *E. coli BGM*; KL: *Klebsiella pneumonia*; PS: *Pseudomonas aeruginosa*; ST: *Staphylococcus aureus*, Rm: roots of *A. P* var. *pyrethrum*; Gm: seeds of *A. P* var. *pyrethrum* (L.); Fm: leaves *A. P* var. *pyrethrum* (L.); Cm: capitula of *A. P* var. *pyrethrum* (L.); Rf: roots of *A. P* var. *depressus* (Ball) Maire; Gf: seeds of *A. P* var. *depressus* (Ball) Maire; Ff: leaves of *A. P* var. *depressus* (Ball) Maire; and Cf: capitula of *A. P* var. *depressus* (Ball) Maire).

Principal component analysis (PCA) is a good tool for data extraction from multivariate matrices and represents the data as a set of a few orthogonal variables [58]. The samples are plotted in red, and the parameters are plotted in blue. In the present work, the first two principal components accounted for 35.90% and 27.93% of the data of the original matrix successively (Figure 2). The first Principal component (PC) is correlated positively with the total phenols and flavonoids, MBC E5, and MBC ST. Therefore, a negative correlation can be observed between the same PC and antioxidant activities as well as the rest of the antibacterial activities (MIC and MBC) except ABTS.

Considering the similarities of the samples, PCA allowed the distinction of three groups: A, B, and C, each of which had similar characteristics in terms of antioxidant and antibacterial activities. The first group (A), composed of Ff and Fm samples, had high total phenolic and flavonoids and, thus, high antioxidant and antibacterial activities in comparison to the other samples. There was a large difference between the Rm and Rf samples; Rm was characterized by a high content in polyphenol and flavonoids and better antibacterial activities compared to the Rf sample, which was characterized by better antioxidant activities.

## 3. Materials and Methods

### 3.1. Plant Material

**Figure 3:**
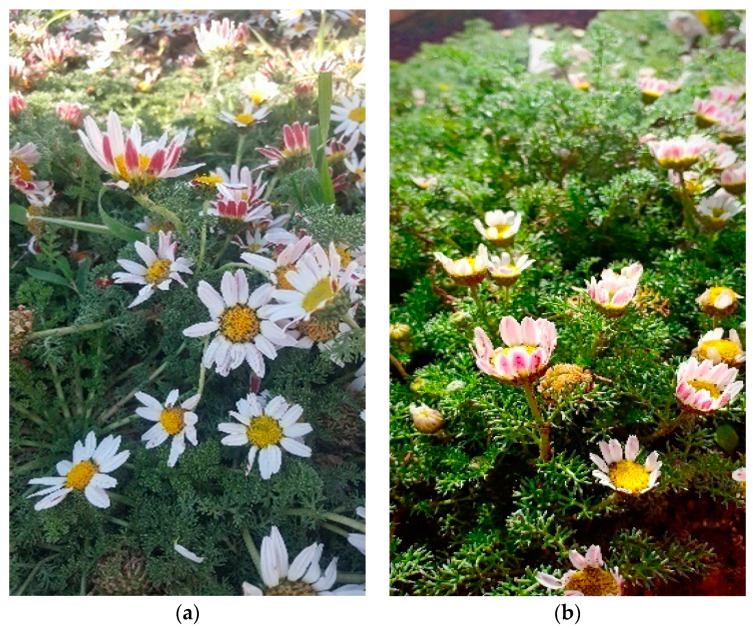
(**a**): *Anacyclus pyrethrum* var. pyrethrum (L.); (**b**)*: Anacyclus pyrethrum* var. depressus (Ball) Maire.

The two *A. pyrethrum* (L.) varieties (Figure 3), *A. P* var. *pyrethrum* (L.) and *A. P* var. *depressus* (Ball) were collected in May 2018 from the Timehdite area (33, 14174573° N5, 15887801° W) at the Middle Atlas from Morocco. The plants were identified by the Botanist Amina Bari and given the voucher specimen no. A31/31-5-18/TM; A32/31-5-18/TM before being deposited at the Herbarium of the Department of Biology, Laboratory of Biotechnology, Environment, Agri-Food, and Health (LBEAS), Faculty of Sciences, Sidi Mohamed Ben Abdellah (USMBA) Fez University Morocco. The different parts (leaves, capitula, seeds, and roots) of the two *A. pyrethrum* (L.) varieties were air-dried in shade at room temperature for two weeks before being pulverized using an electric grinder and conserved in the laboratory until further use.

### 3.2. Preparation of Extracts

The extracts were prepared by maceration as follows; a total of 100 g of each plant powder was extracted using 1000 mL with 70% ethanol for 24 h. Afterward, the mixtures were filtrated and concentrated under reduced pressure and low temperature. The crude extracts obtained were labeled as follows before being saved until further use away from light: Rm: roots of *A. P* var. *pyrethrum* (L.); Gm: seeds of *A. P* var. *pyrethrum* (L.); Fm: leaves *A. P* var. *pyrethrum* (L.); Cm: capitula of *A. P* var. *pyrethrum* (L.); Rf: roots of *A. P* var. *depressus* (Ball) Maire; Gf: seeds of *A. P* var. *depressus* (Ball) Maire; Ff: leaves of *A. P* var. *depressus* (Ball) Maire; and Cf: capitula of *A. P* var. *depressus* (Ball) Maire.

### 3.3. Extraction Yield

After extraction, the yield was calculated using the formula conceptualized by Falleh et al. [59]:

$$R\;\left(\%\right)\;=\frac{m}{m_{0}}\times \;100$$

where R (%) is the yield expressed as a%, m is the mass in grams of the recovered dry extract, and m_0_ is the mass in grams of the initial plant material.

### 3.4. Phytochemical Characterization

The silylation of different samples was performed according to the protocol reported by Kabran and al. [60]. Briefly, a total of 10 g of each sample was treated with 50 mL of 2 N HCl and petroleum ether before heating for two hours. After cooling, the residues were treated again with 3 × 50 mL of ethyl acetate. The fractions obtained were dried on anhydrous MgSO4 before being concentrated under a vacuum. Next, 200 μL of the *N*-methyl-*N*-trimethylsilyltrifluoroacetamide (MSTFA) agent was added to the fraction obtained and was then heated at 37 °C for a further 30 min; 0.1 μL of the sample was injected into the GC-MS apparatus for analysis using Brand Agilent Technologies Model 5973. Helium was used as a carrier to perform analysis with a pressure range (psi) of 0.9 mL/s. The injector and detector temperatures were set to 250 °C and 260 °C, respectively. The oven temperature was set to 60–300 °C for 10 °C/min and then maintained at 300 °C for 20 min.

### 3.5. Mineral Analysis

The dosage of oligo-elements (Ca, K, Mg, Na, P, Cl, Cl, Cu, Fe, Zn, and Se) was carried out by the regal water method as described in earlier protocols [61].

### 3.6. Determination of Total Content of Phenolic Compounds

The content of phenolic compounds in extracts was determined by the Folin–Ciocalteu method [62], and the results obtained were expressed in mg gallic acid equivalent/g (mg EAG/g).

### 3.7. Determination of Flavonoid Contents

The assessment of flavonoid content was quantified using the method described by Miguel et al. [63], and the results obtained were expressed in quercetin equivalent/g (QE/g).

### 3.8. Estimation of Antioxidant Capacity by Phosphomolybdate Assay (TAC)

The total antioxidant capacity was estimated using the method described by Hamada et al. [64], and the results were expressed in mg ascorbic acid equivalent/g (mg AA/g extract).

### 3.9. Evaluation of Antioxidant Activity

The antioxidant capacity of extracts from the *A. pyrethrum* (L.) varieties was assessed using three different methods: the scavenging activity of DPPH radical as reported by Hamada et al. [65], the scavenging activity ABTS radical (2,2-azino-bis 3-ethylbenzothiazolin-6-sulfonic acid) [63], and the reducing power [66]. Butylated hydroxytoluene (BHT), Trolox, and ascorbic acid were used as standards. The results of each test were given in IC_50_ value (the sample concentration required to scavenge 50% of free radicals).

### 3.10. Evaluation of Antibacterial Activities

Extracts from different parts of *A. P* var. *pyrethrum* (L.) and *A. P* var. *depressus* (Ball) Maire were tested against five microbial strains: one gram-positive (*Staphylococcus aureus*) and four gram-negative (*E. coli (ATB: 57) B6N*, *E. coli (ATB: 97) BGM*, *Pseudomonas aeruginosa*, and *Klebsiella pneumonia*). Strains were all delivered by the laboratory of bacteriology at Hassan II University Hospital Center of Fez. The inoculum suspension was obtained by isolating colonies from 24-h cultures and adjusted to have turbidity close to that of McFarland 0.5 (equivalent to 1–5 × 10^8^ CFU/mL) [67].

#### 3.10.1. The Agar Diffusion Method

The antimicrobial activity of extracts was determined by the agar diffusion method as described by Imtara et al. with some modifications [67]. In short, sterile discs with a diameter of 6 mm were aseptically impregnated with extracts (200 mg/mL) and then placed on Petri dishes inoculated with 100 μL pre-prepared bacterial culture before being incubated for 24 h at 37 °C. Amoxicillin and streptomycin were used as positive controls, and the sterile distilled water was used as a negative control. The tests were triplicated, and the results were given by measuring the inhibition zone (mm) of bacterial growth around the discs.

#### 3.10.2. The Minimum Inhibitory Concentration (MIC) and Minimum Bactericidal Concentration (MBC)

The well plate-based method was used to determine the Minimum Inhibitory Concentration (MIC) and Minimum Bactericidal Concentration (MBC) [54]. A serial dilution of the extracts was performed in a well plate; each well was inoculated with 20 μL of bacterial suspension and 20 μL of extract. The mixture was incubated at 37 °C for 24 h. To the suspension, 1 mg/mL of 2,3,5-triphenyltetrazolium chloride (TTC) was added into each well to indicate bacterial growth. Afterward, 1 mL of the incubated solution with a defined MIC was cultured and conserved at 37 °C for 24 h. The MIC was defined as the lowest concentration of the extract that inhibited bacterial growth, while the concentration which exhibited no bacterial growth was considered the MBC value.

### 3.11. Statistical Analysis

The statistical analyses were performed by Pearson’s correlation coefficient (r) at a significance level of 99% (*p* < 0.01). The data preprocessing and the PCA were accomplished using MultBiplot64 running in MATLAB R2017a.

## 4. Conclusions

The present study revealed that different parts of *Anacyclus pyrethrum* var. *pyrethrum* (L.) and *Anacyclus pyrethrum* var. *depressus* (Ball) Maire are rich in phytochemicals that may be involved in the antioxidant and antibacterial properties investigated in this work. In brief, the information presented here could serve as a valuable database for further research to exploit new natural substances present in different parts of the two varieties to fight free radical damage, and fungal and bacterial infections.

## Figures and Tables

**Table 1:** Aspects, colors, and yields of extracts from *A. P* var. *pyrethrum* (L.) and *A. P* var. *depressus* (Ball) Maire.

*A. P* var. *pyrethrum*			
Parts	Aspects	Colors	Yield
Roots	Pasty	Brown	16%
Leaves	Pasty	Green	14%
Seeds	Pasty	Brown	10%
Capitula	Pasty	Brown	14%
***A. P* var. *depressus***			
**Parts**	**Aspects**	**Colors**	**Yield**
Roots	Pasty	Brown	13%
Leaves	Pasty	Green	11%
Seeds	Pasty	Brown	10%
Capitula	Pasty	Brown	6%

**Table 2:** Chemical compositions of different parts (roots, seeds, leaves, and capitula) of the two varieties *Anacyclus pyrethrum* var. *pyrethrum* (L.) and *Anacyclus pyrethrum* var. *depressus* (Ball).

N°	RT	*m*/*z* Quasi-Molecular Peak	Structural Formula	Compounds	% Area							
					*A. P* var. *pyrethrum*				*A. P* var. *depressus*			
					Roots (Rm)	Seeds (Gm)	Leaves (Fm)	Capitula (Cm)	Roots (Rf)	Seeds (Gf)	Leaves (Ff)	Capitula (Cf)
1	4.35	231(M + H)+	C15H19NO	(2,4)-*N*-isobutyl-2,4-undecadiene-8,10-diynamide	0.97	0.76	2.29	-	4.84	2.20	7.07	-
2	4.92	246(M)+	C16H25ON	*N*-isobutyl-dodeca-2,4,8,10-tetraenamide	6.79	4.44	9.45	15.91	14.14	11.41	9.15	0.57
3	6.12	241(M)+	C9H14F3NO3	Sarcosine, *N*-(trifluoroacetyl)-, butyl ester	2.65	0.65	4.26	-	6.08	4.88	11.99	-
4	6.44	153(M)+	C4H3N5S	Thiadiazolo[5,4-d]pyrimidin-7-amine	-	-	-	-	0.74	-	-	-
5	6.98	193(M + H)+	C12H17ON	*N*-isobutyl-2,4-octadiene-6-monoynamide	0.76	0.68	0.68	0.74	0.69	2.04	0.82	-
6	7.71	116(M)+	C5H8O3	Levulinic acid	37.47	50.45	7.01	3.66	15.90	14.84	17.74	30.01
7	8.43	104(M)+	C3H4O4	Malonic Acid	8.48	6.39	16.86	6.50	6.98	11.50	8.42	-
8	8.77	177(M)+	C11H15ON	*N*-isobutyl-2,4-heptadiene-6-monoynamide	-	1.52	6.29	-	-	-	-	-
9	9.38	256(M)	C16H32O2	Palmitic Acid	2.85	2.75	3.17	8.34	5.30	7.29	3.44	0.77
10	9.65	285(M)+	C17H19NO3	Morphinan-6-One, 4,5α-Epoxy-3-Hydroxy-17-Methyl	2.17	1.31	2.86	4.93	2.21	2.00	1.65	-
11	10.62	147(M + H)+	C9H8O2	Cinnamic acid	-	-	10.53	-	-	-	-	-
12	10.75	278(M + H)+	C18H31NO	2,4)-undecadiene-8,10-diyne-*N*-tyramide	11.09	5.34	16.50	46.07	23.61	13.32	14.15	1.58
13	11.64	271(M)+	C18H25NO	*N*-isobutyl-dodeca-2,4,8,10-tetraenamide (Anacycline)	2.94	8.63	-	-	-	0.62	0.69	64.27
14	12.12	221(M)+	C14H23NO	*N*-isobutyl-2,6,8-decatrienamide	0.63	-	2.06	-	2.01	-	3.36	-
15	12.61	223(M)+	C14H25NO	(2E,4E)-*N*-(2-methylpropyl)deca-2,4-dienamide (Pellitorine)	1.16	6.04	0.78	-	2.73	13.21	2.49	-
16	12.94	274(M + H)+	C18H27NO	Tetradeca-2E-diny-8,10-diynoic acid IBA	0.77	-	0.59	2.72	3.61	1.28	-	-
17	13.39	270(M + H)+	C18H23NO	Tetradeca-2E,4E, nE-trienoic-8,10-diynoic acid IBA	-	2.85	1.86	-	-	6.61	-	-
18	13.67	102(M)+	C5H10O2	Isovaleric acid	1.28	4.13	4.14	-	0.74	0.75	1.30	-
19	13.80	243(M)+	C16H21NO	*N*-isobutyl-2,4-undecadiene-8,10-diynamide	-	-	-	-	2.41	-	1.95	-
20	14.10	313(M)+	C20H27NO2	Dodeca-2E,4E, nE-trienoic acid 4-hydroxyphenylethylamide	3.54	1.15	1.58	2.08	2.01	1.27	3.78	-
21	14.57	251(M)+	C16H29NO	2, 8)-*N*-isobutyl-2,8-dodecadienamide	0.87	-	1.19	1.19	0.96	-	1.97	0.75
22	15.15	341(M)+	C22H31NO2	Tetradeca-2E,4E,8Etrienoic acid 4-hydroxyphenylethylamide	0.61	-	0.82	-	1.06	-	2.06	-

**Table 3:** Mineral content in different parts (leaves, capitula, roots, and seeds) of *A. P* var. *pyrethrum* (L.) and *A. P* var. *depressus* (Ball) Maire mg/Kg dry matter.

		Ca mg/kg	Cu mg/kg	Fe mg/kg	K mg/kg	Mg mg/kg	Mn mg/kg	Na mg/kg	P mg/kg	Bi mg/kg	Se mg/kg	Zn mg/kg
***A. P* var. *pyrethrum* (L.)**	Roots (Rm)	38,643	96.36	5221.8	17,239	4956.66	129.12	610.88	31,577	1.19	20.45	487.45
	Capitula (Cm)	23,995	136.8	4071.9	18,709	4982.64	102.64	280.05	14,252	<1	20.28	152.65
	Seeds (Gm)	35,031	1490.6	34,645	14,908	4920.43	294.79	600.65	61,582	<1	<1	437.35
	Leaves (Fm)	88,529	29.12	6948.2	12,955	4921.91	263.88	221.46	9411.7	2.97	20.58	185.52
***A. P* var. *depressus* (Ball) Maire**	Roots (Rf)	88,515	42.84	5333.7	6893.4	4879.92	157.26	188.96	9554.2	4.64	19.76	180.87
	Capitula (Cf)	29,437	349.33	9476	16,266	4968.11	137.91	578.88	12,954	3.55	16,59	165.62
	Seeds (Gf)	45,492	2171.4	38,371	6139.3	4921.92	305.8	507.49	36,385	<1	<1	377.98
	Leaves (Ff)	88,533	302.7	12,414	7259.1	4888.24	236.3	191.17	5145.4	<1	14.05	174.36

**Table 4:** Total phenolic, flavone, and flavonol contents of different extracts of *A. P* var. *pyrethrum* (L.) and *A. P* var. *depressus* (Ball) Maire.

	Samples	Total Phenolic (mg GAE/g)	Flavone and Flavanol Content (mg QE/g)
***A. P* var. *pyrethrum*** **(L.)**	Roots (Rm)	25.96 ± 1.93	0.88 ± 0.02
	Capitula (Cm)	26.46 ± 1.19	5.47 ± 0.07
	Seeds (Gm)	44.05 ± 1.84	2.40 ± 0.03
	Leaves (Fm)	51.78 ± 0.49	13.53 ± 0.05
***A. P* var. *depressus* (Ball) Maire**	Roots (Rf)	5.44 ± 0.21	2.40 ± 0.02
	Capitula (Cf)	15.21 ± 1.19	3.47 ± 0.07
	Seeds (Gf)	38.44 ± 2.19	3.88 ± 0.24
	Leaves (Ff)	38.75 ± 2.91	9.57 ± 0.02

Values are expressed as means ± SD.

**Table 5:** The antioxidant activities (DPPH (2,2-diphenyl-1-picrylhydrazyl), ABTS (2,2-azino-bis 3-ethylbenzothiazolin-6-sulfonic acid), FRAP(ferric reducing antioxidant power), and TAC (total antioxidant capacity)) of different extracts from *A. P* var. *pyrethrum* (L.) and *A. P* var. *depressus* (Ball) Maire.

	Samples	TAC (mg AA/g)	DPPH (IC50 = mg/mL)	ABTS (IC50 = mg/mL)	Reducing Power (IC50 = mg/mL)
***A. P* var. *pyrethrum***	Roots (Rm)	708.74 ± 11.63	0.18 ± 0.005	0.14 ± 0.001	1.19 ± 0.005
	Capitula (Cm)	203.00 ± 3.84	0.16 ± 0.013	0.07 ± 0.001	1.08 ± 0.013
	Seeds (Gm)	577.84 ± 4.65	0.05 ± 0.0003	0.05 ± 0.0008	0.49 ± 0.0004
	Leaves (Fm)	508.45 ± 9.77	0.04 ± 0.001	0.03 ± 0.0004	0.38 ± 0.005
***A. P* var. *depressus***	Roots (Rf)	542.16 ± 4.88	0.07 ± 0.0007	0.05 ± 0.001	0.38 ± 0.005
	Capitula (Cf)	160.33 ± 10.23	0.08 ± 0.023	0.05 ± 0.0009	0.59 ± 0.007
	Seeds (Gf)	521.77 ± 4.88	0.04 ± 0.001	0.05 ± 0.001	0.25 ± 0.008
	Leaves (Ff)	238.77 ± 5.35	0.03 ± 0.0007	0.03 ± 0.0002	0.43 ± 0.010
**Standard**	BHT	-	0.009 ± 0.0001	-	-
	Trolox	-	-	0.019 ± 0.003	-
	Ascorbic acid	-	-	-	0.003 ± 0.001

Values are expressed as means ± SD.

**Table 6:** The diameter of inhibition zone (DI = mm), the minimum inhibitory concentration (MIC = mg/mL), and minimal bactericidal concentration (MBC = mg/mL) of different extracts from *A. P* var. *pyrethrum* (L.) and *A. P* var. *depressus* (Ball) Maire.

			*E. coli (ATB: 57) B6N*	*E. coli (ATB: 97) BGM*	*Staphylococcus aureus*	*Pseudomonas aeruginosa*	*Klebsiella pneumonia*
***A. P* var. pyrethrum (L.)**	Roots (Rm)	DI (mm)	ND	6.6 ± 0.84	ND	5.5 ± 0.70	6.1 ± 0.14
		MIC (mg/mL)	50	25	0.78	50	25
		MBC (mg/mL)	200	100	6.25	200	100
	Capitula (Cm)	DI (mm)	7.5 ± 0.70	8.25 ± 0.35	15 ± 0	15.65 ± 0.91	8.5 ± 0.70
		MIC (mg/mL)	25	100	1.56	50	50
		MBC (mg/mL)	100	200	6.25	200	200
	Seeds (Gm)	DI (mm)	9.3 ± 0.42	6.35 ± 0.49	7.5 ± 0.70	5.65 ± 0.91	10 ± 1.41
		MIC (mg/mL)	50	50	50	50	50
		MBC (mg/mL)	100	200	200	200	200
	Leaves (Fm)	DI (mm)	11.85 ± 0.21	7.2 ± 0.28	ND	9.35 ± 0.49	15.3 ± 0.42
		MIC (mg/mL)	25	50	12.5	25	25
		MBC (mg/mL)	200	200	100	100	100
***A. P* var. depressus (Ball) Maire**	Roots(Rf)	DI (mm)	ND	ND	7 ± 0	11 ± 0	5.75 ± 0.35
		MIC (mg/mL)	50	50	0.39	50	50
		MBC (mg/mL)	200	200	12.5	200	200
	Capitula (Cf)	DI (mm)	ND	9.5 ± 0.70	12.5 ± 0.70	11.1 ± 0.14	9 ± 0
		MIC (mg/mL)	25	100	0.78	50	50
		MBC (mg/mL)	100	200	3.13	200	200
	Seeds (Gf)	DI (mm)	ND	8.5 ± 0.70	7 ± 0	7.8 ± 0.28	7.5 ± 0.70
		MIC (mg/mL)	100	50	25	25	50
		MBC (mg/mL)	100	200	200	200	200
	Leaves (Ff)	DI (mm)	ND	5.5 ± 0.70	ND	8.25 ± 0.35	11 ± 0
		MIC (mg/mL)	25	25	1.56	25	25
		MBC (mg/mL)	200	100	25	100	200
**Control positive**	Ampicillin	DI (mm)	ND	ND	ND	8.5 ± 0.70	ND
		MIC (mg/mL)	ND	ND	0.001	ND	ND
		MBC (mg/mL)	ND	ND	0.001	ND	ND
	Streptomycin	DI (mm)	10.9 ± 0.14	ND	30.7 ± 0.98	16.65 ± 0.49	30.75 ± 1.06
		MIC (mg/mL)	0.25	0.5	0.06	ND	0.003
		MBC (mg/mL)	0.5	0.5	0.06	ND	0.003
**Control negative**	Ethanol	DI, MIC and MBC	ND	ND	ND	ND	ND
	D.W	DI, MIC and MBC	ND	ND	ND	ND	ND

Values are expressed as means ± SD.

## Data Availability

All data reported here is available from the authors upon request.

## Abbreviation

*A. P* var. *pyrethrum*	*Anacyclus pyrethrum* var. *pyrethrum* (L.)
*A. P* var. *depressus*	*Anacyclus pyrethrum* var. *depressus* (Ball)
Rm	*Anacyclus pyrethrum* var. *pyrethrum* roots
Gm	*Anacyclus pyrethrum* var. *pyrethrum* seeds
Fm	*Anacyclus pyrethrum* var. *pyrethrum* leaves
Cm	*Anacyclus pyrethrum* var. *pyrethrum* capitula
Rm	*Anacyclus pyrethrum* var. *depressus* roots
Gm	*Anacyclus pyrethrum* var. *depressus* seeds
Fm	*Anacyclus pyrethrum* var. *depressus* leaves
Cm	*Anacyclus pyrethrum* var. *depressus* capitula
MIC	Minimum Inhibitory Concentration
MBC	Minimum Bactericidal Concentration
